# The Apple Doesn’t Fall Far from the Tree? Paranoia and Safety Behaviours in Adolescent-Parent-Dyads

**DOI:** 10.1007/s10802-023-01128-y

**Published:** 2023-09-23

**Authors:** Sven N. Schönig, Elizabeth Thompson, Jessica Kingston, Brandon A. Gaudiano, Lyn Ellett, Katarina Krkovic

**Affiliations:** 1 Department of Clinical Psychology & Psychotherapy, Universität Hamburg, Hamburg, Germany; 2 Department of Psychiatry and Human Behavior, Brown University, Providence, Rhode Island, USA; 3 Department of Psychology, Royal Holloway, University of London, Surrey, UK; 4 School of Psychology, University of Southampton, Southampton, UK

**Keywords:** Paranoia, Delusions, Adolescence, Safety-seeking behaviours, Psychosis, Parents

## Abstract

Paranoia is a common experience in adolescence that may entail the use of safety behaviours (e.g. avoidance), which are assumed to maintain paranoia in the long run. As the development of paranoia and related safety behaviours in youth may be influenced by their caregivers, we aimed to investigate the associations of paranoia and safety behaviours in adolescents and their parents. Adolescents from the general population aged 14–17 and one of their parents (*N* = 142 dyads) were recruited via Qualtrics to complete online surveys including measures of paranoia, safety behaviour use, anxiety, and demographics. We fitted an Actor-Partner-Interdependence Model (APIM) for testing dyadic parent–child interaction by using structural equation modelling and controlled for adolescents’ and parents’ anxiety. Results indicated that paranoia positively predicted safety behaviour use in adolescents and in parents. There were significant positive intra-dyad (i.e. parent-adolescent) correlations for both paranoia and safety behaviour use. One partner effect was significant: parental paranoia positively predicted the safety behaviour use of their adolescent child. Conversely, adolescents’ paranoia did not predict their parents’ safety behaviour use. Our findings corroborate prior research demonstrating an association between paranoia and safety behaviours among adults, and extend this association to adolescents. Children of parents experiencing paranoia are at increased risk of developing paranoia and safety behaviours, which indicates the need for interventions that target paranoia and safety behaviours in family systems.

**Supplementary Information:**

The online version contains supplementary material available at 10.1007/s10802-023-01128-y.

Paranoia describes the fixed belief that others intend harm, which is not shared by most others, and not amenable to change despite conflicting evidence (American Psychiatric Association, 2013). It is distributed along a continuum ranging from mild, subclinical forms that are common in the general population to more severe forms that manifest in psychotic disorders (van Os et al., 2009). Research indicates that the trajectories that lead to the formation of clinical paranoia in adulthood may already commence in youth. For example, adolescents are at a high risk of experiencing paranoia, with around 20%-30% reporting frequent paranoid thoughts (Bird et al., 2019; Wigman et al., 2011). Although these experiences are transitory and not pathological for most of those affected, the frequency of paranoid thoughts is strongly associated with distress and reduced well-being in adolescence (Wigman et al., 2011). Moreover, distress from these subclinical experiences has been linked to increased risk for psychosis (Nelson et al., 2022; Rekhi et al., 2019). Examining the mechanisms that contribute to the maintenance of paranoia in adolescence could therefore help prevent the exacerbation into fully developed delusions or other psychiatric conditions in adulthood.

While some factors implicated in the maintenance of paranoia in adults have also been found to be relevant to paranoia in adolescents (e.g., depression, anxiety, low self-esteem, affective reactivity, and sleep difficulties; Bird et al., 2017), many other factors have not yet been investigated in youth. One such potential maintenance factor put forward in several theoretical models of paranoia are safety behaviours (Beck, 2008; Freeman, 2016; Morrison, 2001). Safety behaviours are actions that aim to prevent or mitigate a perceived threat (Salkovskis, 1991). They most often manifest as avoidance of certain situations (e.g. not using public transportation) or using defensive measures in the situation (e.g. carrying a pocket knife) (Freeman et al., 2001, 2007). Originally investigated in anxiety research, the concept of safety behaviours also found application in psychosis research, as safety behaviours are assumed to maintain anxious and paranoid threat beliefs in a similar way. In particular, avoidant safety behaviours reduce the chances of being confronted with disconfirmatory evidence (e.g., avoiding social situations altogether prevents the experience that others can be friendly instead of threatening) and the absence of the feared outcome (e.g., being attacked by strangers) is attributed to the use of the safety behaviours instead of one’s inaccurate threat expectation (e.g., “I was not attacked by strangers only because I avoided eye contact with them”) (Freeman, 2016; Salkovskis, 1991).

Evidence acquired in clinical adult samples indicates that safety behaviours are positively associated with threat beliefs in psychotic disorders (Tully et al., 2017) and experimental research supports the assumption that they act as a causal factor in the maintenance of paranoia (Freeman et al., 2016; Pot-Kolder et al., 2018). In non-clinical adult samples, avoidant safety behaviours have been shown to be common behavioural reactions to paranoia (Ellett et al., 2003) and paranoia was strongly associated with self-reported safety behaviour use (Simpson et al., 2012). However, to date, only two studies have investigated the occurrence of paranoia and safety behaviours in youth: A recent qualitative study by Bird et al. (2022) demonstrated the presence of safety behaviours (e.g. avoidance of social situations, defensive strategies) in adolescents experiencing elevated levels of paranoia, but did not provide a quantitative assessment of this experience. In another study, Bird et al. (2019) found a moderate positive association between paranoia and safety behaviours in a non-clinical adolescent population. Yet, this finding is limited by the study’s narrow focus on social-media-related safety behaviours that do not fully portray the wider spectrum of safety behaviours in daily life. Therefore, it is still undetermined whether the association of paranoia and safety behaviours can be replicated when employing a broader conceptualisation of safety behaviours.

In addition to intraindividual maintenance processes, adolescents’ experience of paranoia might also be influenced by interindividual processes. Research indicates that adolescents’ paranoia is associated with parenting variables (e.g., overprotectiveness, abuse, and lack of care; Brown et al., 2021) and that parental stress and (low) warmth moderates the effect of adverse life events on adolescents’ paranoia (Kingston et al., 2023). Therefore, it appears likely that parents are influential for the development of paranoia and paranoia-related behavioural reactions in adolescents. This might trace back to parents’ own paranoia: Research suggests a genetic liability for both clinical and subclinical paranoia, indicating that adolescents may inherit a predisposition for paranoia from their parents (Taylor et al., 2022). Conversely, learning processes could lead children, who witness the paranoia of a parent, to adopt their beliefs about hostility and threat in the environment and vulnerability of the self. In consequence, parents’ paranoia might transmit to their children via multiple pathways. Perlman et al. (2022) have highlighted the relevance of dyadic parent–child interaction dynamics for the transmission of anxiety, and while there is evidence for the transmission of depression and anxiety symptoms from parents to adolescents (Johnco et al., 2021), evidence for an association between parents’ paranoia and adolescents’ paranoia in the general population is currently lacking.

In response to the experience of paranoia, children might develop safety behaviours by imitating those they observe in their parents. Similar transmission processes of anxious behavioural reactions have been documented in anxiety disorders: Children of anxious parents can acquire safety behaviours such as avoidance through observational learning (Aktar et al., 2017). There is a dearth of research, however, exploring links between adolescents’ and parents’ safety behaviours in the context of paranoia. Moreover, as avoidant behaviours can also be learned from parents’ verbal expression of threat beliefs (Aktar et al., 2017), learning could also take place without any observable display of safety behaviours by parents. Therefore, parental paranoid beliefs could represent another cause of adolescents’ safety behaviours. Conversely, adolescents’ paranoia could also affect the safety behaviours of their parents. However, while studies have shown that some forms of psychopathology (e.g. depressive symptoms) can transmit from children to parents (Johnco et al., 2021; Pérez-Edgar et al., 2021), there is currently no evidence suggesting that behavioural responses to paranoia are related to the paranoia of one’s offspring.

Based on the presented literature suggesting a pathway to the development of paranoia and safety behaviours in adolescence via the transmission of parental beliefs and behaviours, we aimed to investigate the association of paranoia and safety behaviours in adolescents and their parents. To this end, we employed the framework of an actor-partner-interdependence model (APIM; Cook & Kenny, 2005), which can be used to model variable associations between individuals that are organised in dyads. We hypothesised that adolescents’ and parents’ paranoia would predict their respective safety behaviour use (actor effects). Moreover, we hypothesised that paranoia and safety behaviours, respectively, would be positively correlated between adolescents and their parents (intra-dyad association). Finally, we hypothesised that parents’ paranoia would predict adolescents’ safety behaviour use (partner effect). As anxiety is a known correlate of safety behaviours (Freeman et al., 2001) and paranoia (Freeman et al., 2012), we wanted to ensure that the hypothesised effect of paranoia on safety behaviours would not be driven by covarying anxiety. We preregistered our hypotheses and analyses prior to the analysis on the Open Science Foundation (OSF) registrations platform (https://osf.io/puct6).

## Method

### Participants

We collected data via Qualtrics, an online survey platform and recruitment service. Recruited dyads were composed of one adolescent aged between 14 and 17 years and one of their parents/carers from the United Kingdom. We used quota sampling, aiming for a balanced gender and age quota in adolescents (50% of sample male/female; 50% of sample aged 14–15 years/16–17 years). Apart from English language proficiency and adolescents’ age range, no further inclusion criteria were specified. Sample size was powered (1-*ꞵ* = 0.90) to detect an effect of *f*^2^ = 0.10 in multiple linear regression.

### Design and Procedure

This study analyses data from an online survey that examined risk factors relating to the development and exacerbation of paranoia. Additional information on the study design and demographic variables is reported in Kingston et al. (2023). We used a cross-sectional dyadic study design and defined paranoia as the independent variable and safety behaviour use as the dependent variable. The study received ethical approval from Royal Holloway, University of London. Recruited participants were Qualtrics users who had indicated that they had an adolescent child. Parents provided informed consent, adolescents provided informed assent, and then both received access to the online survey consisting of several questionnaires, though not all are reported in this study. Adolescents and parents completed the same subset of questionnaires in the current study. Participants were required to respond to all items to progress in the survey. We implemented two attention check items throughout the survey to filter inattentive response patterns. Qualtrics checked all collected data for indicators of fraudulent or low-quality survey completion (i.e., duplicates, bots, straightlining responses, speeding through the survey, mismatch between geolocated IP address and stated location). Flagged responses were manually reviewed by Qualtrics and removed if necessary. Qualtrics also removed participants who declined consent or had incomplete data. Participants were monetarily compensated for their time.

### Measures

#### Revised Green et al. Paranoid Thoughts Scale (R-GPTS; Freeman et al., 2021)

The R-GPTS is an 18-item self-report instrument that assesses paranoid thoughts during the last month on two dimensions: ideas of reference and persecution. Items are rated on a 5-point Likert scale from 0 = "Not at all" to 4 = "Totally". We used the 10-item persecution subscale as it better captures the perceived intentionality of paranoid ideation and relates more strongly to persecutory delusions (Freeman et al., 2021). The persecution subscale demonstrated excellent internal consistency in our sample (*α*_*adolescents*_ = *α*_*parents*_ = 0.96) consistent with Freeman et al. (2021). The R-GPTS has been validated in samples of adults (Freeman et al., 2021) and adolescents (Schlier et al., 2023) from the general population.

#### Measure of Safety Behaviours (MSB)

First and last author participated in the development of the MSB, a novel 14-item questionnaire, to assess safety behaviour use in relation to paranoia during the last month (Online Resource 1). Items were partially adapted from existing measures of safety behaviours such as the Safety Behaviours Questionnaire (SBQ; Freeman et al., 2001) and the Measure of Common Responses to psychosis (MCR; Tully et al., 2017). All items of the MSB are preceded with "To protect myself from other people, danger, or threat, …" and then describe a safety behaviour, e.g. "… I avoided personal contact or eye contact with other people". Items are rated on a 7-point Likert scale from 0 = "Not at all" to 6 = "Very much", and summed up to a total score. We aimed to obtain first evidence of the convergent and discriminant validity of the MSB with a supplemental analysis (Online Resource 2). To this end, we calculated correlations with related constructs (paranoia, maladaptive emotion regulation, perceived neighbourhood safety), and with diverging constructs (anxiety and adaptive emotion regulation) and compared them using the R-package cocor (Diedenhofen & Musch, 2015). The results of this psychometric evaluation provide preliminary support for the convergent and discriminant validity of the MSB (Online Resource 2). The MSB demonstrated excellent internal consistency in the present sample (*α*_*adolescents*_ = *α*_*parents*_ = 0.94).

#### Depression Anxiety Stress Scales (DASS-21; Parkitny & McAuley, 2010)

Anxiety was measured using the 7-item anxiety subscale of the DASS-21. The DASS-21 is a 21-item short form of the original DASS that assesses depression, anxiety and stress via three subscales. Items are rated on a 4-point Likert scale from 0 = "Did not apply to me at all" to 3 = "Applied to me very much, or most of the time". The DASS-21 anxiety subscale demonstrated good internal consistency in our sample (α_adolescents_ = 0.88; α_parents_ = 0.86) that was comparable to its validation study (Osman et al., 2012). The DASS-21 has been validated in samples of adolescents (Evans et al., 2021) and adults (Osman et al., 2012).

### Data Analysis

We used the Actor-Partner-Interdependence Model (APIM; Cook & Kenny, 2005) to test our hypotheses. The APIM is an analysis method used to model interdependent variable relationships in dyadic data, i.e. in cases where members of a dyad are assumed to influence each other. Fitting an APIM estimates “actor” and “partner” effects of a predictor variable on an outcome variable. In our study, paranoia was the predictor variable and safety behaviour use was the outcome variable. Actor effects describe the influence of paranoia on safety behaviour use for one actor (e.g., adolescents) when controlling for the partners’ (e.g., parents’) paranoia. Partner effects describe the influence of the partners’ paranoia on the actors’ safety behaviour use when controlling for the actors’ paranoia. To further rule out a potential confounding effect of anxiety on safety behaviour use, we included anxiety as a covariate in all APIM analyses.

We conducted APIM analysis for distinguishable dyads using the online app APIM_SEM (Stas et al., 2018), which uses the R-package lavaan (Rosseel, 2012) and maximum likelihood estimation to fit an APIM structural equation model. We assumed that adolescents and parents were theoretically distinguishable and could have distinct actor and partner effects, but chose to test this assumption statistically by comparing the model for distinguishable dyad members with the model for indistinguishable dyad members using a χ^2^-test (Kenny et al., 2006). APIM analysis relies on the assumption of nonindependence, which we tested by calculating the correlation between both dyad members’ outcome variables, as recommended by Cook and Kenny (2005). In the main analyses, we report partial correlation coefficients for the respective intra-dyad associations of paranoia (H1) and safety behaviours (H2). For actor (H3, H4) and partner effects (H5) of paranoia on safety behaviours and the effect of the covariate anxiety on safety behaviours, we report unstandardised (*b*) and standardised (*β*) effect estimates. In accordance with effect size conventions for linear relationships (Cohen, 1988), we considered *β* = 0.10 a small effect,* β* = 0.30 a medium effect, and *β* = 0.50 a large effect. We conducted all significance tests with α = 0.05. All p-values for APIM path coefficients were adjusted for multiple testing using Bonferroni-Holm correction.

## Results

### Sample Characteristics

Three hundred and twenty-five adult participants with an adolescent child started the online survey and consented to participate. Of those, 156 adolescent-parent dyads completed the survey. Fourteen dyads were removed by Qualtrics for not passing data quality checks (i.e., their data were indicative of fraudulent or low-quality responses and were excluded after manual review). This resulted in a final sample of *N* = 142 adolescent-parent dyads. Descriptive statistics for demographic and clinical variables are reported in Table 1. Most adolescent participants attended school and were in U.K. school years 8–13 (90.8%). Parents were predominantly married (59.9%), and had completed A-level-equivalent or tertiary education (73.2%). Paranoia levels were within average range (R-GPTS persecution ≤ 5; Freeman et al., 2021) for 70.4% of adolescents and 64.8% of parents. “Severe” or “very severe” paranoia (R-GPTS persecution ≥ 18), indicative of likely persecutory delusions according to Freeman et al. (2021), was reported by 10.5% of adolescents and 13.3% of parents.

**Table 1:** Sample Characteristics for Adolescents’ Subsample and Parents’ Subsample

	Adolescents (*n* = 142)	Parents (*n* = 142)
Age *M* (*SD*)	15.40 (1.09)	43.91 (7.38)
Gender *n* (*%*)		
Female	78 (54.9)	107 (75.4)
Male	62 (43.7)	35 (24.6)
Other	1 (0.7)	-
Trans Male	1 (0.7)	-
Ethnicity *n* (*%*)		
Asian/Asian British	3 (2.1)	5 (3.5)
Black/African/Caribbean/Black British	2 (1.4)	2 (1.4)
Mixed/Multiple ethnic group	7 (4.9)	1 (0.7)
Other ethnic group	1 (0.7)	1 (0.7)
White	129 (90.8)	133 (93.7)
Clinical Measures *M* (*SD*)		
DASS Anxiety	2.13 (3.32)	4.44 (4.59)
MSB	19.44 (19.68)	22.75 (19.57)
R-GPTS Persecution	5.52 (8.68)	6.70 (9.16)

*DASS* Depression Anxiety Stress Scales, *MSB* Measure of Safety Behaviours, *R-GPTS* Revised Green et al. Paranoid Thought Scale

### Actor-Partner Interdependence Model

The test for distinguishability of dyad members was significant (χ^2^ (12) = 141.58, *p* < 0.001), indicating that the model for indistinguishable dyad members and the model for distinguishable dyad members were statistically different and that the latter model better fit the data. Adolescents’ and parents’ safety behaviours were positively correlated (*r* = 0.63, *p* < 0.001), indicating that the assumption of nonindependence required for APIM analysis was met (Cook & Kenny, 2005).

**Fig. 1:**
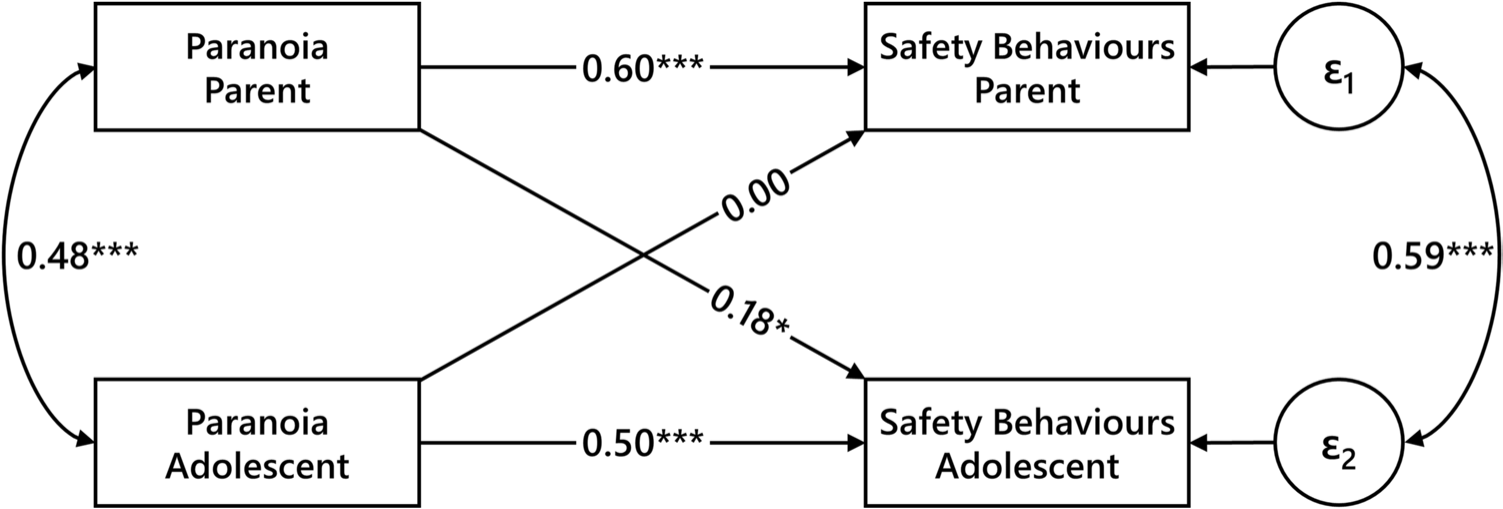
Actor-Partner Interdependence Model for the Effect of Paranoia on Safety Behaviours. *Note.* Values on top of single-headed arrows are standardised beta coefficients. Values next to double-headed arrows are partial correlation coefficients. *ε*_i_ denote error terms. The covariate anxiety is included in the model, but not pictured for simplicity. *p < 0.05; **p < 0.01; ***p < 0.001

The APIM structural equation model converged after 132 iterations and fit indices indicated good model fit (χ^2^(2) = 1.08, *p* = 0.58, *CFI* = 1.00, *TLI* = 1.02, *RMSEA* = 0.00, 90% *CI* [0.00;0.14], *SRMR* = 0.01). In the model, 47.5% of the variance in adolescents’ safety behaviour use and 45.1% of the variance in parents’ safety behaviour use was explained. Results of the APIM analysis are shown in Fig. 1.

Both paranoia (*r* = 0.48, *p* < 0.001) and safety behaviour use (*r* = 0.59, *p* < 0.001) showed a significant positive intra-dyad (i.e. parent-adolescent) correlation. In line with our hypotheses, both actor effects were significant: paranoia positively predicted safety behaviour use with a large effect in adolescents (*b* = 1.13, *β* = 0.50, *SE* = 0.18, *p* < 0.001) and a large effect in parents (*b* = 1.28, *β* = 0.60, *SE* = 0.17, *p* < 0.001). For a paranoia value of zero, the predicted score (intercept) for adolescents’ safety behaviour use was equal to 9.01 (*p* < 0.001) and for parents’ safety behaviour use equal to 12.11 (*p* < 0.001).

As hypothesised, one partner effect was significant: parental paranoia positively predicted the safety behaviour use of their adolescent child with a small effect (*b* = 0.39, *β* = 0.18, *SE* = 0.15, *p* = 0.03). Thus, for every increase of one unit in parents’ paranoia total score, adolescents reported an increase of 0.39 on the safety behaviours scale. This was above and beyond the effect of adolescents’ paranoia on their respective safety behaviour use. Conversely, adolescents’ paranoia did not predict their parents’ safety behaviour use (*b* = -0.01, *β* = -0.004, *SE* = 0.16, *p* = 0.96).

### Covariate analysis

Adolescents’ anxiety was significantly correlated with adolescent paranoia (*r* = 0.61, *p* < 0.001), parental paranoia (*r* = 0.29, *p* = 0.005) and parental anxiety (*r* = 0.40, *p* < 0.001), but did not predict adolescents’ safety behaviour use (*b* = 0.72, *β* = 0.12, *SE* = 0.37, *p* = 0.16). Parental anxiety was significantly associated with parental paranoia (*r* = 0.64, *p* < 0.001) and adolescent paranoia (*r* = 0.37, *p* < 0.001), but did not predict parental safety behaviour use (*b* = 0.48, *β* = 0.11, *SE* = 0.28, *p* = 0.18).

## Discussion

This study set out to examine paranoia and safety behaviours in dyads composed of adolescents from the general population and one of their parents. In line with our preregistered hypotheses, we found that paranoia predicted safety behaviour use for both adolescents and their parents. Moreover, adolescents’ and parents’ paranoia as well as safety behaviour use were associated with each other. Lastly, we found that parental paranoia predicted the safety behaviour use of their adolescent child. Additional findings showed that anxiety was associated with paranoia in adolescents and parents but did not predict safety behaviours.

In our study, paranoia experienced by adolescents and parents during the last month predicted their respective safety behaviour use with a large effect. Hence, our findings add to the evidence from previous studies showing an association between paranoia and safety behaviours. In a non-clinical adult sample, Simpson et al. (2012) reported an association of *r* = 0.55, which is comparable in size to our findings. Moreover, our results are in line with Bird et al. (2019), who found paranoia to predict social media-related safety behaviours in adolescents. By assessing a broader range of safety behaviours, we were able to expand on these findings and demonstrate an association between paranoia and more general safety behaviour use in adolescents. Our results further indicate that the association of threat beliefs and safety behaviours found in adult samples with psychotic disorders (Tully et al., 2017) is also present in the adult and adolescent general population. Our sample included a wide spectrum of paranoia severity, with 10–13% of participants experiencing severe or very severe paranoia. This percentage is comparable to the prevalence of severe or very severe paranoia reported in a previous study (7% in adults without mental health problems; 17% in adults with non-psychotic mental health problems; Freeman et al., 2021). Regardless of severity, experiencing paranoia therefore appears to be associated with attempts to protect against the assumed threat. This is consistent with aetiological continuity, the notion that maintenance mechanisms relevant to clinical manifestations of psychotic symptoms also translate to subclinical manifestations as both are distributed along the same continuum (Myin-Germeys et al., 2003).

Furthermore, this study is to our knowledge the first to demonstrate an association between adolescents’ and parents’ paranoia in a sample from the general population. Our results therefore expand research demonstrating the essential role of parents for the formation and maintenance of paranoia in youth. In addition to studies showing that paranoia is associated with parenting behaviours (Brown et al., 2021; Kingston et al., 2023; Rankin et al., 2005; Udachina & Bentall, 2014) and the quality of the relationship with parents (Riggio & Kwong, 2011), we were able to provide evidence that adolescents’ self-reported paranoia is directly associated with their parents’ self-reported paranoia. While part of this association is likely due to hereditary factors, which previous research has shown explain around 40% of the variance in adolescent paranoia, the influence of environmental factors has been shown to outweigh the genetic influence on paranoia (Taylor et al., 2022). It is therefore probable that our findings also reflect the result of learning processes that may explain the transmission of paranoia from parents to their children. Parents who experience paranoia might verbally express their threat beliefs and display overprotective parenting behaviours that imply the presence of threat to their children who then acquire paranoid beliefs themselves. This explanation is in line with the well-established association of parental overprotection and paranoia (Brown et al., 2021; Rankin et al., 2005; Udachina & Bentall, 2014; Valiente et al., 2014). However, the learning processes with regard to paranoia within families are yet to be examined.

Our results further showed that both parents’ paranoia and parents’ safety behaviour use were associated with the safety behaviour use of their adolescent child. Notably, this was above and beyond the influence of adolescents’ own paranoia and anxiety. This could indicate that the extent to which adolescents try to protect themselves against assumed threat is not only affected by their own beliefs, but also their parents’ beliefs and behaviours. Research on anxiety disorders has established that parents’ anxiety can contribute to the acquisition of avoidant (safety) behaviours in their children via verbal and non-verbal learning pathways (Aktar et al., 2017). Although this can only be speculated on the basis of our results, similar processes could be at play in paranoia. Parents’ expression of paranoia might therefore imply the need to protect against threat and lead children to use safety behaviours. Moreover, children may learn these safety behaviours from their parents. The strong association between parents’ safety behaviour use and adolescents’ safety behaviour use could imply that adolescents acquire a repertoire of safety behaviours that is similar to that of their parents. This could take place via observational learning processes. It remains to be explored whether children only acquire a general tendency to protect against threat and consequently develop idiosyncratic safety behaviours or whether they learn to imitate their parents’ safety behaviours. Future research should investigate this by assessing the concordance of safety behaviour types used by parents and their children.

Some limitations are worth mentioning. This study was cross-sectional and thus, we cannot infer temporal precedence with respect to paranoia and safety behaviours, nor can we determine causality with respect to parents’ variables influencing adolescent paranoia and/or safety behaviours. While we investigated paranoia as a predictor of safety behaviours, this does not necessarily represent the variables’ true relationship. Our findings are open to the possibility that safety behaviour use predicts paranoia or that a bidirectional relationship exists. Experimental studies are needed to explore causality and longitudinal studies could provide further insight into how these experiences develop and impact real-world functioning and clinical outcomes over time. Moreover, given the nature of this online study, which included only self-report data, we were unable to verify the identities and demographics of participants. We also did not assess whether dyad members were biologically related to each other or whether they resided in the same household. Moreover, we could not evaluate the clinical significance of experiences such as paranoia, safety behaviours, and anxiety. In particular, it is unknown whether participants’ endorsement of items aiming to measure paranoia and safety behaviours was based on experiences of (sub)clinical paranoia, experiences of genuine threat, or a combination of both. Hence, we cannot rule out that participants’ paranoia scores may have been associated (and potentially confounded) with exposure to environmental threats such as bullying, as has been found in previous research (Bird et al., 2019; Kingston et al., 2023). Another limitation is the preliminary evidence for the psychometric quality of the MSB. Further use of the MSB seems promising due to its excellent reliability and our supplemental analyses provided support for the convergent and discriminant validity of the MSB. However, more extensive validation is required to further substantiate this. Furthermore, our sample was mostly White and most parents were female and educated, which reduces the generalisability of our results. Future studies should aim to collect data from more diverse and representative samples of parents and children.

Taken together, our findings support the idea that safety behaviours may serve as a modifiable treatment target for interventions in adults and adolescents who experience paranoia. Dropping safety behaviours is considered crucial to the cognitive-behavioural treatment of anxiety disorders (Blakey & Abramowitz, 2016; Helbig-Lang & Petermann, 2010) and paranoia interventions have begun to adopt this approach (Freeman et al., 2021; Pot-Kolder et al., 2018). Furthermore, the fact that parents’ and adolescents’ variables were strongly associated with each other yields another clinical implication: Paranoia and safety behaviours appear to “run” in families, which suggests that intervention and prevention strategies should also aim to address the whole family system instead of an isolated individual. As our findings show, adolescents who show safety behaviours in response to paranoia are likely to have parents who also show safety behaviours. To prevent adolescents from developing rigid safety behaviours that contribute to the maintenance of paranoia, modifying the family environment that reproduces such safety behaviours seems essential. Family interventions could provide targeted support for vulnerable adolescents who are exposed to parents’ paranoia and safety behaviours, but also help parents find behavioural strategies and communication strategies that promote trust in their children.

### Supplementary Information

Below is the link to the electronic supplementary material.

Supplementary file1 (DOCX 21.7 KB)

Supplementary file2 (DOCX 26.7 KB)

## Data Availability

The dataset can be requested using this link https://osf.io/8m3u2.

## References

[CR1] Aktar, E., Nikolic, M., & Bögels, S. M. (2017). Environmental transmission of generalized anxiety disorder from parents to children: Worries, experiential avoidance, and intolerance of uncertainty. *Dialogues in Clinical Neuroscience*, *19*(2), 137–147. 10.31887/dcns.2017.19.2/eaktar 10.31887/DCNS.2017.19.2/eaktar PMC5573558 28867938

[CR2] American Psychiatric Association. (2013). *Diagnostic and statistical manual of mental disorders* (5th ed.). 10.1176/appi.books.9780890425596

[CR3] Beck AT (2008). Schizophrenia.

[CR4] Bird JC, Evans R, Waite F, Loe BS, Freeman D (2019). Adolescent Paranoia: Prevalence, Structure, and Causal Mechanisms. Schizophrenia Bulletin.

[CR5] Bird JC, Freeman D, Waite F (2022). The journey of adolescent paranoia: A qualitative study with patients attending child and adolescent mental health services. Psychology and Psychotherapy: Theory, Research and Practice.

[CR6] Bird JC, Waite F, Rowsell E, Fergusson EC, Freeman D (2017). Cognitive, affective, and social factors maintaining paranoia in adolescents with mental health problems: A longitudinal study. Psychiatry Research.

[CR7] Blakey SM, Abramowitz JS (2016). The effects of safety behaviors during exposure therapy for anxiety: Critical analysis from an inhibitory learning perspective. Clinical Psychology Review.

[CR8] Brown P, Waite F, Freeman D (2021). Parenting behaviour and paranoia: A network analysis and results from the National Comorbidity Survey-Adolescents (NCS-A). Social Psychiatry and Psychiatric Epidemiology.

[CR9] Cohen, J. (1988). *Statistical power analysis for the behavioral sciences* (2nd ed.). Lawrence Erlbaum Associates.

[CR10] Cook WL, Kenny DA (2005). The actor-partner interdependence model: A model of bidirectional effects in developmental studies. International Journal of Behavioral Development.

[CR11] Diedenhofen, B., & Musch, J. (2015). cocor: A comprehensive solution for the statistical comparison of correlations. *PLoS One, 10*(4), e0121945. 10.1371/journal.pone.0121945 10.1371/journal.pone.0121945 PMC4383486 25835001

[CR12] Ellett L, Lopes B, Chadwick P (2003). Paranoia in a nonclinical population of college students. Journal of Nervous and Mental Disease.

[CR13] Evans L, Haeberlein K, Chang A, Handal P (2021). Convergent validity and preliminary cut-off scores for the anxiety and depression subscales of the DASS-21 in US adolescents. Child Psychiatry & Human Development.

[CR14] Freeman D (2016). Persecutory delusions: A cognitive perspective on understanding and treatment. The Lancet Psychiatry.

[CR15] Freeman D, Bradley J, Antley A, Bourke E, DeWeever N, Evans N, Černis E, Sheaves B, Waite F, Dunn G, Slater M, Clark DM (2016). Virtual reality in the treatment of persecutory delusions: Randomised controlled experimental study testing how to reduce delusional conviction. British Journal of Psychiatry.

[CR16] Freeman, D., Garety, P. A., & Kuipers, E. (2001). Persecutory delusions: Developing the understanding of belief maintenance and emotional distress. *Psychological Medicine*, *31*(7), 1293–1306. 10.1017/S003329170100455X 10.1017/s003329170100455x 11681555

[CR17] Freeman D, Garety PA, Kuipers E, Fowler D, Bebbington PE, Dunn G (2007). Acting on persecutory delusions: The importance of safety seeking. Behaviour Research and Therapy.

[CR18] Freeman, D., Loe, B. S., Kingdon, D., Startup, H., Molodynski, A., Rosebrock, L., Brown, P., Sheaves, B., Waie, F., & Bird, J. C. (2021). The revised Green et al., Paranoid Thoughts Scale (R-GPTS): Psychometric properties, severity ranges, and clinical cut-offs. *Psychological Medicine*, *51*(2), 244–253. 10.1017/s0033291719003155 10.1017/S0033291719003155 PMC7893506 31744588

[CR19] Freeman D, Stahl D, McManus S, Meltzer H, Brugha T, Wiles N, Bebbington P (2012). Insomnia, worry, anxiety and depression as predictors of the occurrence and persistence of paranoid thinking. Social Psychiatry and Psychiatric Epidemiology.

[CR20] Helbig-Lang S, Petermann F (2010). Tolerate or eliminate? A systematic review on the effects of safety behavior across anxiety disorders. Clinical Psychology: Science and Practice.

[CR21] Johnco CJ, Magson NR, Fardouly J, Oar EL, Forbes MK, Richardson C, Rapee RM (2021). The role of parenting behaviors in the bidirectional and intergenerational transmission of depression and anxiety between parents and early adolescent youth. Depression and Anxiety.

[CR22] Kenny DA, Kashy DA, Cook WL (2006). Dyadic data analysis.

[CR23] Kingston, J. L., Ellett, L., Thompson, E. C., Gaudiano, B. A., & Krkovic, K. (2023). A Child–Parent Dyad Study on Adolescent Paranoia and the Influence of Adverse Life Events, Bullying, Parenting Stress, and Family Support. *Schizophrenia Bulletin*, sbad119. 10.1093/schbul/sbad119 10.1093/schbul/sbad119 PMC10686324 37621256

[CR24] Morrison AP (2001). The interpretation of intrusions in psychosis: An integrative cognitive approach to hallucinations and delusions. Behavioural and Cognitive Psychotherapy.

[CR25] Myin-Germeys I, Krabbendam L, van Os J (2003). Continuity of psychotic symptoms in the community. Current Opinion in Psychiatry.

[CR26] Nelson, B., Yuen, H. P., Amminger, G. P., Berger, G., Chen, E. Y., de Haan, L., ... & McGorry, P. D. (2022). Distress Related to Attenuated Psychotic Symptoms: Static and Dynamic Association With Transition to Psychosis, Nonremission, and Transdiagnostic Symptomatology in Clinical High-Risk Patients in an International Intervention Trial. *Schizophrenia Bulletin Open*, *3*(1), 1–9. 10.1093/schizbullopen/sgaa006 10.1093/schizbullopen/sgaa006 PMC11205873 39144772

[CR27] Osman A, Wong JL, Bagge CL, Freedenthal S, Gutierrez PM, Lozano G (2012). The Depression Anxiety Stress Scales—21 (DASS-21): Further Examination of Dimensions, Scale Reliability, and Correlates. Journal of Clinical Psychology.

[CR28] Parkitny L, McAuley J (2010). The Depression Anxiety Stress Scale (DASS). Journal of Physiotherapy.

[CR29] Pérez-Edgar K, LoBue V, Buss KA (2021). From parents to children and back again: Bidirectional processes in the transmission and development of depression and anxiety. Depression and Anxiety.

[CR30] Perlman SB, Lunkenheimer E, Panlilio C, Pérez-Edgar K (2022). Parent-to-child anxiety transmission through dyadic social dynamics: A dynamic developmental model. Clinical Child and Family Psychology Review.

[CR31] Pot-Kolder RMCA, Geraets CNW, Veling W, van Beilen M, Staring ABP, Gijsman HJ, Delespaul PAEG, van der Gaag M (2018). Virtual-reality-based cognitive behavioural therapy versus waiting list control for paranoid ideation and social avoidance in patients with psychotic disorders: A single-blind randomised controlled trial. The Lancet Psychiatry.

[CR32] Rankin P, Bentall R, Hill J, Kinderman P (2005). Perceived relationships with parents and paranoid delusions: Comparisons of currently ill, remitted and normal participants. Psychopathology.

[CR33] Rekhi G, Rapisarda A, Lee J (2019). Impact of distress related to attenuated psychotic symptoms in individuals at ultra high risk of psychosis: Findings from the Longitudinal Youth at Risk Study. Early Intervention in Psychiatry.

[CR34] Riggio HR, Kwong WY (2011). Paranoid thinking, quality of relationships with parents, and social outcomes among young adults. Journal of Family Issues.

[CR35] Rosseel, Y. (2012). lavaan: An R package for structural equation modeling. *Journal of Statistical Software*, *48*, 1–36. 10.18637/jss.v048.i02

[CR36] Salkovskis PM (1991). The Importance of Behaviour in the Maintenance of Anxiety and Panic: A Cognitive Account. Behavioural Psychotherapy.

[CR37] Schlier, B., Ellett, L., Thompson, L., Gaudiano, B., Krkovic, K., & Kingston, J. L. (2023). Measuring Paranoid Beliefs in Adolescents: A Comparison of the Revised-Green et al.’s Paranoid Thoughts Scale (R-GPTS) and the Bird Checklist of Adolescent Paranoia (B-CAP). [Manuscript submitted for publication]. 10.1007/s10802-024-01187-9 PMC11289251 38568405

[CR38] Simpson J, MacGregor B, Cavanagh K, Dudley RE (2012). Safety Behaviours, Rumination and Trait Paranoia in a Non-Clinical Sample. Journal of Experimental Psychopathology.

[CR39] Stas L, Kenny DA, Mayer A, Loeys T (2018). Giving dyadic data analysis away: A user-friendly app for actor–partner interdependence models. Personal Relationships.

[CR40] Taylor MJ, Freeman D, Lundström S, Larsson H, Ronald A (2022). Heritability of psychotic experiences in adolescents and interaction with environmental risk. JAMA Psychiatry.

[CR41] Tully S, Wells A, Morrison AP (2017). An exploration of the relationship between use of safety-seeking behaviours and psychosis: A systematic review and meta-analysis. Clinical Psychology and Psychotherapy.

[CR42] Udachina A, Bentall RP (2014). Developmental pathway to paranoia is mediated by negative self-concept and experiential avoidance. Psychosis.

[CR43] Valiente C, Romero N, Hervas G, Espinosa R (2014). Evaluative beliefs as mediators of the relationship between parental bonding and symptoms of paranoia and depression. Psychiatry Research.

[CR44] van Os J, Linscott RJ, Myin-Germeys I, Delespaul PA, Krabbendam L (2009). A systematic review and meta-analysis of the psychosis continuum: Evidence for a psychosis proneness-persistence-impairment model of psychotic disorder. Psychological Medicine.

[CR45] Wigman JTW, Vollebergh WAM, Raaijmakers QAW, Iedema J, Van Dorsselaer S, Ormel J, Verhulst FC, Van Os J (2011). The structure of the extended psychosis phenotype in early adolescence - A cross-sample replication. Schizophrenia Bulletin.

