# Supplementary material for: The Apple Doesn’t Fall Far from the Tree? Paranoia and Safety Behaviours in Adolescent-Parent-Dyads
Source: Res Child Adolesc Psychopathol. 2023 Sep 23;52(2):267–75. doi: 10.1007/s10802-023-01128-y (PMC10834552; doi:10.1007/s10802-023-01128-y)
Supplement: Supplementary file 1 — Supplementary file1 (DOCX 21.7 KB) [file 10802_2023_1128_MOESM1_ESM.docx]

# Measure of Safety Behaviours (MSB)

This part addresses how you have dealt with thoughts and feelings of threat. The questionnaire below lists a few possible reactions. People who feel threatened by others sometimes act in one or more of the following ways to protect themselves. Please indicate for each statement whether you behaved or acted this way in the last month.
To protect myself from other people, danger or threat, ...

|  | 0  Not at all | 1 | 2 | 3 Some-what | 4 | 5 | 6 Very much |
| --- | --- | --- | --- | --- | --- | --- | --- |
| ...I avoided an activity, a place or a situation. |  |  |  |  |  |  |  |
| ...I avoided personal contact or eye contact with other people. |  |  |  |  |  |  |  |
| ...I escaped from a situation in a hurry. |  |  |  |  |  |  |  |
| ...I carried certain objects with me that make me feel safer. |  |  |  |  |  |  |  |
| ...I distracted myself |  |  |  |  |  |  |  |
| ...I was vigilant and alert. |  |  |  |  |  |  |  |
| ...I got angry or aggressive towards other people. |  |  |  |  |  |  |  |
| ...I tried to get help from people I know or the police. |  |  |  |  |  |  |  |
| ...I tried not to attract attention to myself. |  |  |  |  |  |  |  |
| ...I went to a safe space. |  |  |  |  |  |  |  |
| ...I researched information. |  |  |  |  |  |  |  |
| ...I carefully observed my surroundings. |  |  |  |  |  |  |  |
| ...I examined everyday objects or food. |  |  |  |  |  |  |  |
| ...I talked to someone about my thoughts. |  |  |  |  |  |  |  |
