# Supplementary material for: The Apple Doesn’t Fall Far from the Tree? Paranoia and Safety Behaviours in Adolescent-Parent-Dyads
Source: Res Child Adolesc Psychopathol. 2023 Sep 23;52(2):267–75. doi: 10.1007/s10802-023-01128-y (PMC10834552; doi:10.1007/s10802-023-01128-y)
Supplement: Supplementary file 2 — Supplementary file2 (DOCX 26.7 KB) [file 10802_2023_1128_MOESM2_ESM.docx]

**Psychometric Evaluation of the Measure of Safety Behaviours (MSB)**

**Supplemental Methods**

**Instruments**

***Short Cognitive Emotion Regulation Questionnaire (CERQ-short; Garnefski et al., 2001)***

We assessed habitual emotion regulation with the CERQ-short to test the convergent and discriminant validity of the MSB. The CERQ-short is a 18-item questionnaire that assesses the frequency of use of adaptive (acceptance, positive refocusing, refocus on planning, positive reappraisal, putting into perspective) and maladaptive (self-blame, rumination, catastrophizing, other-blame) emotion regulation strategies on a Likert scale from 1 = “(Almost) Never” to 5 = ”(Almost) Always”. The CERQ has been validated in adults (Garnefski et al., 2006) and adolescents (Garnefski et al., 2001).

***Perceived Neighbourhood Safety***

We assessed adolescents’ (but not parents’) perceived safety in the neighbourhood with two items (“How safe do you feel out alone in your neighbourhood during the day?”; “How safe do you feel out alone in your neighbourhood during the night?”; rated on a scale from 1 – 4) to help validate the MSB. We expected that a reduced feeling of safety in the neighbourhood would be associated with a higher MSB total score, i.e. stronger safety behaviour use.

**Data Analysis**

Since the MSB was developed to assess putatively maladaptive safety-seeking responses specifically tied to paranoia, we calculated correlations between the MSB total score and the R-GPTS persecution subscale, and between the MSB and the CERQ maladaptive subscale, as an indication of convergent validity. This was done using the full sample. Additionally, for adolescents only, we calculated the correlation between the MSB and perceived neighbourhood safety (this variable was not assessed in parents). To examine discriminant validity, we tested whether the MSB was more strongly correlated with the R-GPTS persecution subscale than with the DASS-21 anxiety subscale, and whether the MSB was more strongly correlated with the CERQ maladaptive subscale than with the CERQ adaptive subscale. These comparisons were calculated using the two-sided test by Hittner et al. (2003) provided in the R-package cocor (Diedenhofen & Musch, 2015).

**Supplemental Results**

**Scale Characteristics**

Means and standard deviations for individual MSB items are presented in Table S1. MSB total scores did not differ significantly between adolescents and parents (*t* = -1.42, |*d*| = 0.17, *p* = .16), or based on gender (*t* = 0.35, |*d*| = 0.04, *p* = .73), age (*r* = .03, *p* = .57), or ethnicity (*F*(4,279) = 0.54, *p* = .71).

**Table S1**

Means and Standard Deviations of the Measure of Safety Behaviours (MSB) Items

| MSB item | Adolescents  (*n* = 142) | Parents  (*n* = 142) |
| --- | --- | --- |
| “To protect myself from other people, danger or threat, …” |  |  |
| 1. I avoided an activity, a place or a situation. | 1.44 (1.90) | 1.92 (1.95) |
| 2. I avoided personal contact or eye contact with other people. | 1.43 (1.79) | 1.82 (1.87) |
| 3. I escaped from a situation in a hurry. | 1.18 (1.68) | 1.65 (1.88) |
| 4. I carried certain objects with me that make me feel safer. | 0.61 (1.38) | 0.87 (1.61) |
| 5. I distracted myself | 1.18 (1.49) | 1.77 (1.80) |
| 6. I was vigilant and alert. | 1.89 (1.94) | 2.47 (2.00) |
| 7. I got angry or aggressive towards other people. | 0.99 (1.57) | 0.96 (1.48) |
| 8. I tried to get help from people I know or the police. | 1.09 (1.79) | 1.03 (1.76) |
| 9. I tried not to attract attention to myself. | 1.70 (2.19) | 1.85 (2.04) |
| 10. I went to a safe space. | 1.68 (2.11) | 1.72 (2.03) |
| 11. I researched information. | 1.30 (1.98) | 1.61 (2.03) |
| 12. I carefully observed my surroundings. | 2.04 (2.12) | 2.30 (2.12) |
| 13. I examined everyday objects or food. | 1.11 (1.91) | 1.28 (1.82) |
| 14. I talked to someone about my thoughts. | 1.80 (2.04) | 1.51 (1.90) |

*Note.* Responses range from 0 – 6 for all items. Reported values are *M (SD).*

**Convergent and Discriminant Validity of the MSB**

The MSB total score was strongly correlated with paranoia (*r* = .61, *p* < .001) and moderately correlated with anxiety (*r* = .48, *p* < .001). This difference was significant (*z* = 3.04, *p* = .002). The MSB showed a large correlation with the CERQ maladaptive subscale (*r* = .52, *p* < .001) and a moderate correlation with CERQ adaptive subscale (*r* = .31, *p* < .001). This difference was also significant (*z* = 3.54, *p* < .001). Adolescents’ MSB score had a moderate negative correlation with perceived neighbourhood safety (*r* = -.32, *p* < .001), indicating more use of safety behaviours the more unsafe they felt.

**Supplemental References**

Garnefski, N., Kraaij, V., & Spinhoven, P. (2001). Negative life events, cognitive emotion regulation and emotional problems. *Personality and Individual Differences*, *30*(8), 1311–1327. https://doi.org/10.1016/S0191-8869(00)00113-6

Garnefski, N., & Kraaij, V. (2006). Cognitive emotion regulation questionnaire–development of a short 18-item version (CERQ-short). Personality and individual differences, *41*(6), 1045–1053.

Hittner, J. B., May, K., & Silver, N. C. (2003). A Monte Carlo Evaluation of Tests for Comparing Dependent Correlations. The Journal of General Psychology, *130*(2), 149–168.
